# Exceptionally Long CDR3H Are Not Isotype Restricted in Bovine Immunoglobulins

**DOI:** 10.1371/journal.pone.0064234

**Published:** 2013-05-22

**Authors:** Stefanie Walther, Claus-Peter Czerny, Ulrike S. Diesterbeck

**Affiliations:** Division of Microbiology and Animal Hygiene, Department of Animal Sciences, Faculty of Agricultural Sciences, Institute of Veterinary Medicine, Georg-August University Göttingen, Göttingen, Germany; University of Rome, Italy

## Abstract

Exceptionally long third complementarity determining regions of the heavy chain (CDR3H) were previously described as a specificity of bovine IgG and IgM immunoglobulins. In addition, the genomic organization of the immunoglobulin heavy chain locus remains to be elucidated with a special focus on the number of variable segments (IGHV). By analyzing the variable regions according to the isotype-specific PCR using cDNA-PCR, we were able to prove the existence of exceptional long CDR3H in all bovine isotypes. The corresponding sequences of three distinct amplicons were grouped according to the length of the CDR3H. Sequences of CDR3H possessed 5 to 10, 12 to 31 or at least 48 amino acid residues. Long and mid-length CDR3H were composed of mainly hydrophilic amino acid residues, while short CDR3H also contained hydrophobic amino acid residues. All sequences with long CDR3H were related to the germline variable segment 10. Using the current genome assembly, *Bos taurus* NCBI build 6.1, the genomic organization of the bovine immunoglobulin heavy-chain locus was analyzed. A main locus was investigated on BTA21. Exons coding for variable, diversity, and joining segments, as well as for the constant regions of different isotypes, were also localized on BTA7, BTA8, and BTA20. Together with the information from unplaced contigs, 36 IGHV were detected of which 13 are putatively functional. Phylogenetic analysis revealed two bovine IGHV families (boVH1, boVH2). Thus, the existence of the two bovine families suggested was demonstrated, where boVH1 comprises all functional segments. This study substantially improves the understanding of the generation of immunoglobulin diversity in cattle.

## Introduction

The generation of antibody diversity in vertebrates is subjected to a sequence of steps such as the recombination of separated germline gene segments for both heavy (V, D, and J) and light (V and J) chains. Furthermore, the imprecise junction of the germline gene segments occurs as a result of nucleotide deletions or additions (N, P), introduced by the terminal deoxynucleotidyl transferase during the recombination process. The assembly of two identical heavy and light chains completes the tetrameric molecule [1], [2], [3], [4]. In addition, somatic hypermutations contribute to antibody diversity – dependent or independent of antigen contact [5], [6], [7]. While these general processes of diversification are very similar in all vertebrate species, considerable differences were found in the available pool of the germline V, D, and J segments. Although humans and mice possess a large pool of VDJ genes [8], livestock such as chicken [9], pigs [10], sheep [11], and cattle [6], [12], [13] are relatively restricted in the generation of combinatorial diversity. Therefore, species-dependent mechanisms dominate the different diversification steps or additional options are employed. For instance, in chicken gene conversion, the use of pseudogene sequences is a frequent post-recombinatorial strategy for the generation of the preimmune antibody repertoire [5], [14]. This mechanism was confirmed for λ-light chains in cattle [15] and is discussed in horses [16].

All heavy-chain isotype classes detected in other mammals were also described for cattle [17], [18], whereas the γ-isotype encompasses three sub-classes, namely γ1, γ2, and γ3 [18], [19]. The bovine IGH locus was assigned to the *Bos taurus* autosome (BTA) 21 [20] and localized on the q23-q24 bands [21] or on the q24 band respectively [22], [23]. An IgM-like chain was assigned to BTA11q23 by hybridization [24], [25], which was supported by the detection of six IGHJ segments on the same chromosome [26]. By screening a bovine BAC and Cosmid library, the genomic organization of the IGHC locus was described, as well as the number of the preceding joining segments (IGHJ). Only two out of six IGHJ were classified as functional – of which only one seems to be involved predominantly in the recombination process [21], [26]. The IGHV itself codes for the complementarity determining regions 1 and 2 (CDR1H, CDR2H) and for the N-terminal part of the complementarity determining region 3 (CDR3H). Bovine IGHV offer a restricted set of genes related to one family (boVH1), which shares homologies to the murine Q52 family and human VHII family. Southern blot analyses indicated one additional IGHV family in the germline repertoire but only expression of boVH1 has been observed yet [6], [12], [13], [27], [28]. The definite number and organization of IGHV remains under further investigation.

Another peculiarity is the organization of the bovine IGHD locus. Ten IGHD genes classified into four families are organized in sub-clusters [29], [30]. A comparison of the IGHD exons revealed huge size differences [29]. Cattle antibodies provide exceptionally long CDR3H consisting of up to 62 amino acid residues (aa) [6], [31], [32], [33], [34], [35]. IGHD2, with 148 bp in size, contributes to those CDR3H and encodes the characteristic hydrophilic Glycine and Tyrosine residues [6], [29], [36]. The high number of Cysteine residues detected is supposed to promote intra-CDR3H disulfide bonds [13]. Mid-length CDR3H – containing one to three Cys residues – were almost always accompanied by one Cys residue found in the CDR2H, which may result in intra CDR disulfide bond formation [31], [37]. The germline encoded IGHV, IGHD, and IGHJ and their imprecise junction during rearrangement cannot fully explain the remarkable length of the CDR3H. Conserved short nucleotide sequences of 13 to 18 nucleotides are specifically inserted into the IGHV and IGHD junction, leading to a further extension of the CDR3H. This mechanism is unique for cattle [30].

To date, these exceptionally long CDR3H have been attributed exclusively to the γ1-3- and µ-isotype [33], [35]. In our study, we demonstrate the expression of exceptionally long CDR3H in all bovine immunoglobulin isotypes. We were able to observe three distinct groups of CDR3H sizes, which were related to their genomic origin. Loci of IGHV were determined on BTA7, BTA21 and seven unplaced contigs.

## Materials and Methods

### *In silico* Analysis of the Bovine IGHV Segments

A sequence search was performed with blastn on *Bos taurus* in the Reference genomic sequences (refseq_genomic) database using the leader and variable region of one mRNA sequence (accession number AY145128). On the identified contigs, the IGHV and their respective leader were annotated together with the recombination signal sequences (RSS). The octamers, TATA boxes, and splicing sites were also noted. The nucleotide sequences of the bovine IGHD1 to 8 and Q52 [30], as well as all IGHJ coding sequences (AY158087, AY149283), were used for a similar alignment approach. The detected IGHV, IGHD, and IGHJ were used in the further analysis of the amplified immunoglobulin sequences. To annotate the constant region locus, IgM (U63637), IgD (AF411240, AF515672), IgG1 (X16701), IgG2 (S82407, X16702), IgG3 (U63638), IgE (AY221098), and IgA (AF109167) bovine coding sequences were applied. Missing transmembrane regions were determined in bovine ESTs (expressed sequence tags). Based on the available sequence data, functionality was defined according to Lefranc [38]. In brief, functional sequences exhibited an open reading frame (ORF) without stop codon, and no defects in the splicing sites, RSS, or in the regulatory elements. If sequence information was missing due to end of contigs or N’s introduced in the sequence but the available sequence offered putative functionality, genes were marked with (F). Classification to ORF included either alterations in the splicing site, RSS, regulatory elements, substitutions of conserved amino acid residues (Cys23, Trp41, Leu89, Cys104 within IGHV or IGHC and a Phe/Trp118-Gly119-X120-Gly121 motif within IGHJ [39]) or orphons ((ORF)). In this case, orphons are located outside of BTA21 [20], [21], [22], [23]. Pseudogenes (Ψ) were characterized by the presence of stop codons or frameshifts. Fragmented loci were also defined as pseudogenes. Functional recombination assays revealed the spacer lengths, the first three nucleotides of the heptamer as well as three consecutive adenosine residues within the nonamer to be crucial for efficient recombination [40], [41].

For the purpose of phylogenetic analysis, the complete nucleotide sequences of bovine IGHV segments were aligned with one member of the human IGHV families 1 to 7, respectively, using the ClustalW algorithm with the ClustalX 2.1 interface [42]. The phylogenetic tree was calculated using the neighbor-joining method, with the exclusion of gaps. The confidence values were compiled with 1000 bootstrap replicates [43]. To root the tree, the sequence of one IGHV segment of the horned shark (accession number X13449) and little skate (X15124) were defined as an outgroup, similar to the method performed by Sitnikova and Su [44] and Almagro et al. [45]. Visualization of the phylogenetic tree was performed using the program NJplot [46].

### Ethical Statement

To collect B-lymphocytes, 20 ml of EDTA blood were taken from the tail vein of a German Simmental bull kept by the Division of Microbiology and Animal Hygiene for demonstrations in claw-treatment within student courses and to study the clinical development of *Mycobacteria avium spp. paratuberculosis* infection. The bull was owned, because he had acquired a natural infection of MAP and showed positive antibody-titers already with an age of 18 months. Similar to other cattle herds, he has to be tested for cattle diseases periodically. The blood sample was taken from the tail vein during regular investigation of infectious diseases in the bull. The plasma was applied e.g. in an indirect ELISA testing for antibodies against *Mycobacteria avium spp. paratuberculosis* or BHV-1. Therefore, no specific approval is required.

### Isolation of PBMCs and cDNA Synthesis

Peripheral blood mononuclear cells (PBMCs) were isolated using Ficoll gradients (GE Healthcare Europe GmbH, Germany) according to the manufacturer’s protocol. Viable B cells were stained with trypan blue and counted. Total RNA was isolated from 1×10^7^ cells using the RNeasy^®^ Mini Kit (Qiagen, Germany). The first-strand cDNA was synthesized using pd(N)_6_ primers from 3 µg of total RNA in a total volume of 20 µl (SuperScript™III First-Strand Synthesis SuperMix, Life Technologies GmbH, Germany).

### Amplification of Immunoglobulin Heavy-chain Isotype Restricted Variable Regions

To amplify the variable region restricted to each isotype, a primer set was generated with one primer hybridizing in the leader region, and individual primers with binding sites in the constant region (CH) of the immunoglobulin heavy chains. The primers were based on database entries and their own sequence information (data not shown). For α, γ1-3, and ε isotypes primers anneal to the CH1. For δ and µ isotypes, primers bind within the CH2 (Table 1). To monitor the integrity and purity of the cDNA, 527 bp of the bovine GAPDH (Glycerinaldehyde 3-phosphate dehydrogenase) were amplified as a positive control. A no template control served as a negative control for the PCR. The total reaction volume of 50 µl included 1 µl of cDNA, 200 µM dNTPs (Bioline, Germany), 5 µl of 10×PCR buffer (75 mM Tris HCl pH 9.0; 2 mM MgCl_2_; 50 mM KCl; 20 mM (NH_4_)_2_SO_4_), 5% DMSO (Dimethyl sulfoxide), 0.4 µM of each primer pair, and 2 units of DNA polymerase (Biotools, Spain). PCR was performed under cycling conditions of 95°C for 5 min, followed by 35 cycles of 95°C for 1 min, 58°C for 1 min, 72°C for 2 min, and terminated with elongation at 72°C for 10 min. The length and purity of the PCR products were evaluated by means of electrophoresis on 1% agarose gels.

**Table 1:** Primer for the isotype-specific amplification of the complete variable regions.

Forward primer	Reverse primer	Primersequence 5′-3′	Approximated product size in bp
BoLH_BACK		ACCCACTGTGGACCCTCCTC	
	BoIgMCH2_FOR	TGCCGTCACCAGAGAGGCTGT	795
	BoIgDCH2_FOR	TGCGTGCTGACCGCCTTGTT	805
	BoIgG1-3CH1_FOR	GGCACCCGAGTTCCAGGTCA	536
	BoIgECH1_FOR	GCCCAGCCTTACACGGGCTT	467
	BoIgACH1_FOR	GCCAGCACGGCAGGGAAGTT	574
GAPDH_for		TGGTCACCAGGGCTGCT	
	GAPDH_rev	GGAGGGGCCATCCACAGTCT	527

One universal forward primer was used for annealing within the leader region. For each isotype, a reverse primer was generated for specific amplification. The annealing sites were selected in the first constant region (IGCH1), with the exception of IgM and IgD. Both isotypes share high homologies in the IGCH1 and therefore, specific reverse primers were generated for binding in the second constant region. The IgG subtypes were not distinguished further. Primers for bovine GAPDH served as cDNA quality control.

### Cloning and Sequencing of the PCR Products

The PCR products were purified and concentrated using the DNA Clean & Concentrator Kit (Zymo Research, USA). Purified products were cloned into the pCR® 2.1-TOPO® 3.9 Kb TA vector (Invitrogen™, Karlsruhe, Germany) and transformed into chemically competent One Shot TOP10 *E. coli* cells (Invitrogen™, Karlsruhe, Germany). Transformants were plated on LB agar containing 0.3 mM ampicillin, 40 µl 2.44 µM X-gal (5-bromo-4-chloro-3-indolyl-beta-D-galactopyranoside), and 40 µl 1 M IPTG (Isopropyl β-D-1-thiogalactopyranoside) for blue/white selection. After incubation at 37°C, overnight cultures of randomly selected white transformants were grown in a 5 ml LB-ampicillin broth. Plasmids were isolated using the MiniPrep Kit (Qiagen, Germany). In order to assess the insert size, plasmid DNA was cleaved with *Eco*RI (New England Biolabs, Germany) and DNA sizes were confirmed by agarose gel electrophoresis.

Twenty plasmids of each PCR product were sequenced according to the chain-termination method [47]. The M13 (-20) Forward and M13 Reverse (Invitrogen, Germany) vector-specific primers, as well as the corresponding gene specific primers, were used for sequencing.

### Nucleotide and Amino Acid Sequence Analyses

The genetic information of the VDJ recombinations was used for further analysis. The amplified part of the constant regions served as verification of the respective isotype. The sequences were analyzed using the DNAStar program (GATC Biotech AG, Germany) and aligned by ClustalW [48].

The deduced amino acid residues of the variable parts were aligned to the IMGT nomenclature [39] using the IMGT/DomainGapAlign [49], [50]. Framework regions, as well as CDRs, were identified and analyzed with regard to their biochemical properties such as the hydrophobicity, polarity, and charge of the amino acid residues incorporated. The CDR3H regions were classified according to their number of amino acid residues. The amino acid compositions of CDR2H and CDR3H were examined for their numbers of Tyr, Gly, aromatic amino acid residues and Cys, since some bovine CDR3Hs are characterized by exceptional length and preferred amino acid residues.

## Results

### Annotation of the Bovine Germline Immunoglobulin Heavy-chain Locus

**Figure 1:**
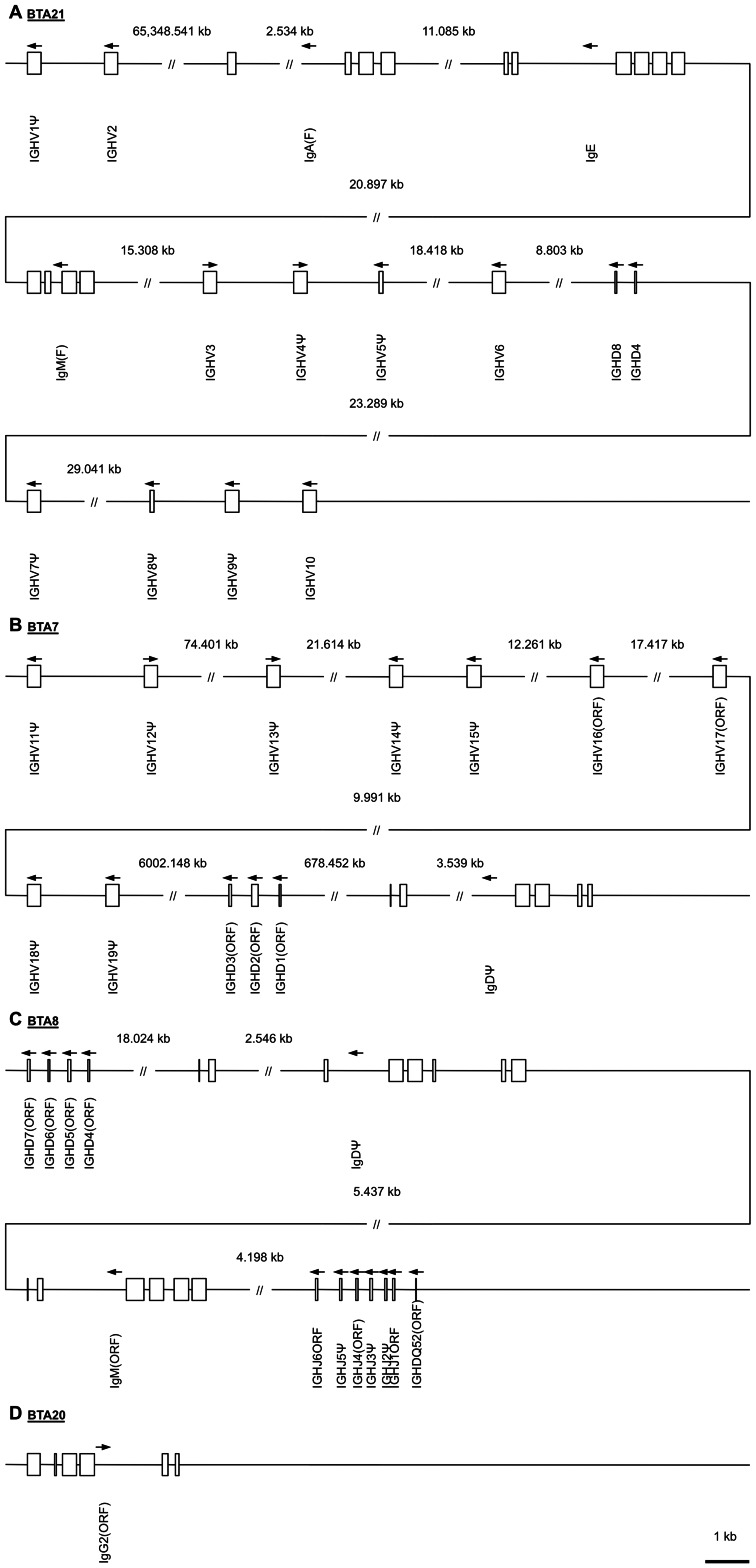
Chromosomal organization of variable (IGHV), diversity (IGHD), joining (IGHJ) segments, and the constant regions of the heavy chains. The physical map displays the order of functional segments (F), pseudogenes (Ψ), and open reading frames (ORF). Classification to “functional” includes an ORF without stop and exhibition of conserved amino acid residues as well as no defects in splicing signals, recombination signal sequences (RSS) or regulatory elements. ORF are defined by alterations in the splicing signals, recombination signal sequences, and/or regulatory elements. In addition, changes to conserved amino acid residues, which may lead to misfolding were included in the ORF classification. Functional elements on orphon localizations are highlighted with ORF in parenthesis (ORF) [38], [39]. Pseudogenes possessed stop codons, frameshifts or mutations of the spacer lengths within the first three nucleotides of the heptamer as well as in three consecutive adenosines residues within the nonamer abolish the recombination [40], [41]. In addition, fragmented loci were also defined as pseudogenes. Arrows indicate the transcription direction.

For the identification of germline IGHV, a search using blastn on all bovine genome assemblies was performed. The contigs NW_003104530.1 and NW_003104538.1 were identified on *Bos taurus* chromosome 21 (BTA21; AC_000178.1). NW_003104530.1 was located at the centromeric region with two IGHV (IGHV1Ψ and IGHV2). A region of about 146 kb on NW_003104538.1, located at the telomeric region, comprised eight IGHV: IGHV3, IGHV4Ψ, IGHV5Ψ, IGHV6, IGHV7Ψ, IGHV8Ψ, IGHV9Ψ, and IGHV10. Upstream of them, the exons coding for the µ, ε and α chains, were identified. Two IGHD (8 and 4) were localized between IGHV6 and IGHV7Ψ (Figure 1A).

The contigs NW_003064289.1, NW_003064290.1, NW_003064296.1, NW_003064297.1, NW_003064298.1, and NW_003064299.1 were localized to the centromeric region on BTA7 (AC_000164.1) involving nine IGHV (IGHV11Ψ, IGHV12Ψ, IGHV13Ψ, IGHV14Ψ, IGHV15Ψ, IGHV16(ORF), IGHV17(ORF), IGHV18Ψ, and IGHV19Ψ). This cluster spans approximately 144 kb. Downstream on BTA7, a cluster of 1131 bp, with exons encoding IGHD1(ORF) to IGHD3(ORF), was identified on contig NW_003064411.1, followed by a pseudogene for a δ chain. The latter one is characterized by fragmented exons 1 and 2, a deleted exon 6 (codes for the secretory region), and frame shifts (Figure 1B).

A third location comprising five IGHD segments, a δ chain pseudogene, a μ chain gene, and six IGHJ segments in about 43 kb was detected on BTA8. Two loci for the IGHD were observed. IGHDQ52(ORF) was the most downstream segment on contig NW_003066919.1, whereas IGHD4(ORF) to 7(ORF) were found to be the most upstream on contig NW_003066918.1 (Figure 1C). Interestingly, BTA20 revealed the genomic information for a γ2 chain (Figure 1D).

In addition, unplaced contigs (NT_182448.1, NT_182449.1, NT_183109.1, NT_185036.1, NT_185907.1, NT_186922.1, and NW_003100762.1) were discovered to contain IGHV segments. Likewise, IGHD1(ORF) and 2 genes were localized on NT_186153.1. NW_001494075.1 includes a gene coding for IGHDQ52 downstream of an IGHJ1 to 6 locus. Genes coding for a μ chain and a δ chain pseudogene were found most upstream in this contig. The contig NW_001503306.1 comprises the genomic information for IGHD4 to 7 and a δ chain pseudogene while IGHD4 and 8 were detected on NW_001504477.2. A locus involving IGHJ4ORF to 6ORF and a putative functional δ chain gene was detected on NT_186572. Additional genomic information for α and ε chains were discovered on NT_185723.1. A genomic order of γ3, γ2, and γ1 was identified on NT_185580.1. Furthermore, a gene coding for γ1 and γ3 was found on NW_003100065.1 and NW_003099305.1, respectively. Along with NW_003100387.1, including a δ chain pseudogene locus, a putative functional δ chain gene was noted on NW_003100112.1.

**Figure 2:**
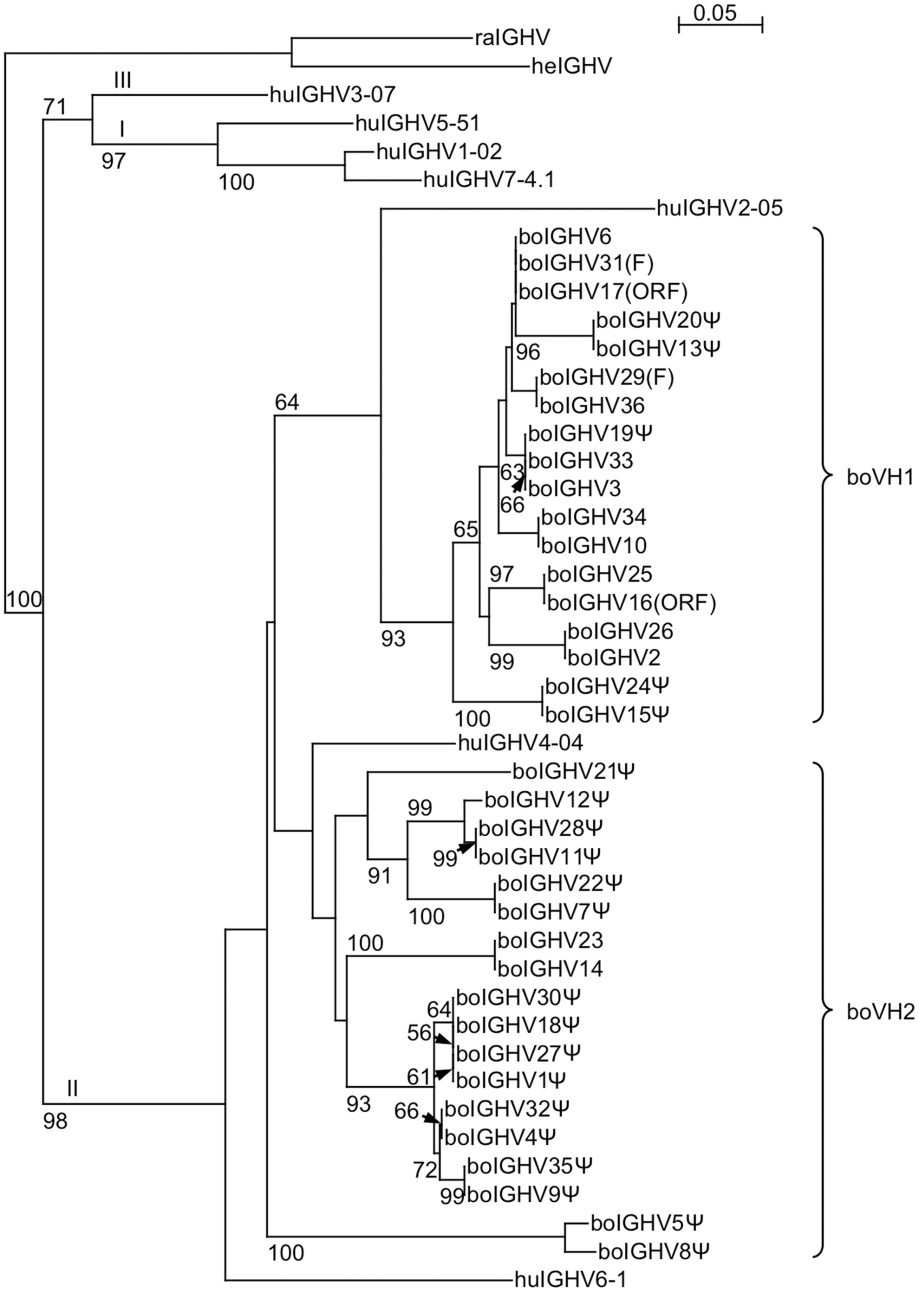
Neighbor-joining phylogenetic tree of the genomic bovine IGHV segments. The complete sequences of the bovine IGHV segment (boIGHV), and one representative sequence of each human family (huIGHV1 to huIGHV7), were used for the comparison. The reliability of the tree was estimated using 1000 bootstrap replicates [43]. Numbers at each node are the percentage bootstrap value and are indicated only when greater than 50%. Arrows mark the respective node. The Roman numerals I, II, and III describe the clans [64]. Two clusters of bovine IGHV were visible and corresponded to two families. The bovine IGHV family 1 (boVH1) comprises all functional segments, whereas boVH2 consists only of pseudogenes. IGHV5Ψ and IGHV8Ψ present fragmented loci, which consist of only 77 bp. They share 70.1% sequence identity with IGHV1Ψ, IGHV18Ψ, IGHV27Ψ, and IGHV30Ψ. We would therefore propose to assign IGHV5Ψ and IGHV8Ψ to boVH2. Horned shark (heIGHV from accession number X13449) and little skate (raIGHV; X15124) represent the outgroup in this analysis, similar to that performed by Sitnikova and Su [44] and Almagro et al. [45]. The scale bar indicates the number of nucleotide substitutions per site.

Thirteen out of the 36 IGHV segments identified are putatively functional (Supplemental Table S1). Eleven IGHV segment pairs shared sequence identity of 100%, namely IGHV3/33, IGHV10/34, IGHV9Ψ/35Ψ, IGHV4Ψ/32Ψ, IGHV7Ψ/22Ψ, IGHV2/26, IGHV1Ψ/27Ψ, IGHV18Ψ/30Ψ, IGHV16(ORF)/25, IGHV14Ψ/23Ψ, and IGHV36/29(F). Since the human IGHV locus has been fully investigated [51], one member of each family was chosen for phylogenetic analysis. The sequences clustered into two distinct branches either with huIGHV2-05 or with huIGHV4-04 and huIGHV6-1. All functional IGHV were phylogenetically related to huIGHV2-05 (Figure 2). This group corresponds to the bovine IGHV family 1 (boVH1) described previously [6], [12], [13]. Multiple alignments revealed two distinct families with identities of at least 80.5% for boVH1 and 79.5% for boVH2. IGHV5Ψ and IGHV8Ψ represent fragmented loci consisting of 77 bp. They share the highest identity of 70.1% with IGHV1Ψ, IGHV18Ψ, IGHV27Ψ, and IGHV30Ψ which are members of boVH2. The highest identity between members of both families was calculated to be 69.7%.

### Amplification of Isotype-specific Variable Regions and Sequence Analyses

**Figure 3:**
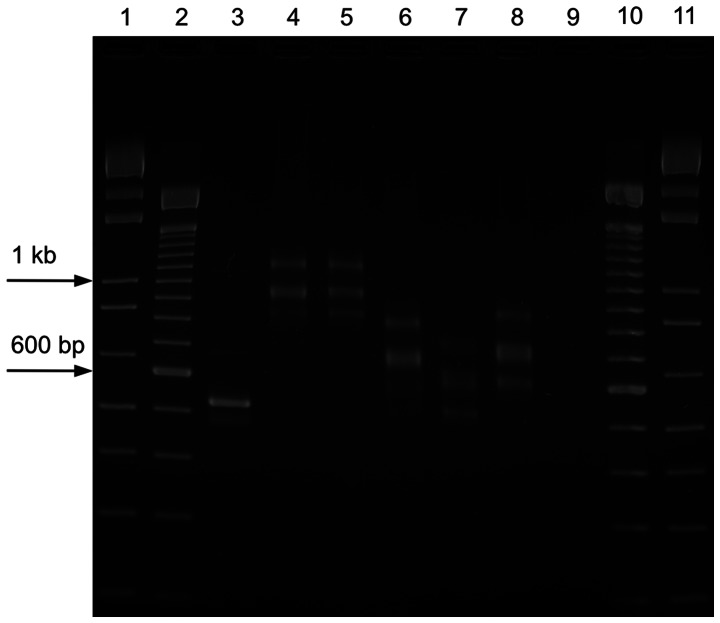
PCR products of the bovine µ, δ, γ1 to 3, ε, and α isotypes. The amplicons of the heavy chain variable domain of each bovine isotype were resolved by 2.5% agarose gel electrophoresis and revealed three distinct products. Lanes 1 and 11∶1kb ladder, lanes 2 and 10∶100 bp ladder. Lane 1: product of the GAPDH positive control; Lanes 4 and 5: in the products of µ and δ, a faint lower band was noticed also with differences in size of 100 bp. The middle and upper bands showed strong amplification. Lanes 6 to 8: the dominant product of isotypes γ1 to 3, ε, and α was observed in the middle with a size difference of about 100 bp compared to both the upper and the lower band. Lane 9 is the no template negative control of the PCR.

The immunoglobulin heavy chains were amplified by PCR for each bovine isotype. Three distinct bands became visible following agarose gel electrophoresis of the amplicons of the µ, δ, γ1 to 3, ε, and α isotypes. The expected product sizes of 467 bp (IgE) to 805 bp (IgD) corresponded with the lowest band. The isotypes γ1 to 3, ε, and α revealed the lowest band, always approximately 100 bp below the middle band, which was again 100 bp smaller than the largest band (Figure 3). The dominant product was observed in the middle band. In contrast, a faint lower band was noticed in the products of IgM and IgD – also with differences in size of 100 bp. The middle and upper bands showed strong amplification. The three bands observed per amplified isotype should allow for grouping of the respective CDR3H lengths. After sub-cloning of the purified products, 20 sequences per isotype were evaluated.

The deduced amino acid sequences were aligned to the IMGT nomenclature for variable domains [39]. The size of the variable regions varied between 111 and 173 amino acid residues (Table 2). The Cysteine residues, forming the intra-chain disulfide bond, were always observed at position 23 and 104. Trp41 and Leu89 were also conserved. These amino acid residue positions are coded by the V segment [39]. A Trp at position 118 and subsequently a Gly119, coded by the J segment, were also found in most sequences analyzed. In four sequences, amino acid residue substitutions were observed at these positions. Two SNPs resulted in the replacement of Gly119 by Asp (SNP: GGC>GAC, in one sequence) or Ser (SNP: GGC>AGC, in two sequences). In one sequence, we found Trp118 substituted by Ser, which was caused by the SNP TTG>TCG.

**Table 2:** Analysis of the complement determining regions (CDR) of the different isotypes.

Isotype	Length of the variable region	Length of the CDR1H	Length of the CDR2H	Length of the CDR3H
IgM	131.65±14.62	8±0	7±0	25.65±14.62
IgD	138.70±17.54	8±0	7±0	32.70±17.54
IgG1-3	138.70±19.39	8±0	7±0	32.70±19.39
IgE	136.55±17.97	8±0	7±0	30.55±17.97
IgA	127.05±12.17	8±0	7±0	21.05±12.17

The average amino acid residue lengths of the complete variable region and the CDR regions, according to the IMGT nomenclature [39], are provided with their standard deviations. The size of the variable regions ranged from 111 to 173 amino acid residues. In total, 20 sequences per isotype were analyzed.

The CDR1H always comprised eight amino acid residues, whereas CDR2H possessed seven amino acid residues. They consisted of both hydrophobic and hydrophilic amino acid residues.

### Composition of the CDR3H with Different Lengths

**Figure 4:**
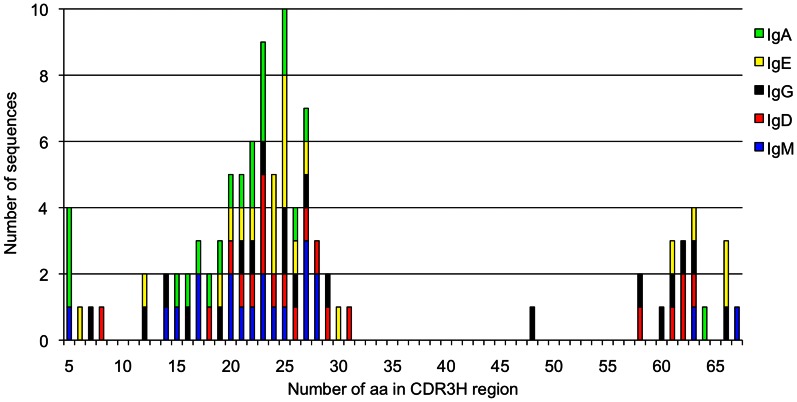
Three distinct sizes of CDR3H in all isotypes. The lengths of CDR3H of all sequences analyzed was divided into three groups. These groups were identified in each isotype, marked in different colors. The numbers of amino acid residues (aa) in CDR3H are indicated on the horizontal axis, whereas the number of sequences possessing each number of aa is provided on the vertical axis.

The three distinct sizes of the CDR3H were noted in all bovine immunoglobulin isotypes. A specific composition of amino acid residues was found within the CDR3H, which seems to correlate with the different lengths of CDR3H. The shortest group (group 1) – comprising five to ten amino acid residues according to Lopez et al. [27] – was characterized by hydrophobic, as well as hydrophilic amino acid residues, without Cysteines (Figure 4). Twelve to 31 amino acid residues formed the middle size (group 2). Within this group, hydrophilic amino acid residues were incorporated predominantly into the CDR3H. The Gly, Tyr, and Cys-rich, long CDR3H included more than 47 amino acid residues (group 3). Gly and Tyr were frequently found within the CDR3H, whereas Gly dominated in most sequences analyzed. Within the exceptionally long CDR3H, four, six, seven or eight Cys were detected, which accumulated in the middle of the CDR3H. Group 1 and 2 only possessed none, one, or two Cys. In two sequences of IgE, with one Cys in the mid-length CDR3H, one additional Cys was found in CDR2H. Gly, Pro and multiple Cys, as well as aromatic residues, were observed in long and intermediate CDR3H.

The IGHV, IGHD, and IGHJ segments were related to their genomic origin by phylogenetic alignment. In general, IGHJ1 was related to all sequences and heavy-chain isotypes were analyzed. Although IGHJ2 is also functional, we did not identify this segment. In addition to the exclusive use of one IGHJ segment, six IGHV segments were recombined. For group 1, segment IGHV3 was detected in six out of seven sequences. One sequence showed IGHV6. The segments IGHV2 (1 sequence), IGHV3 (5 sequences), IGHV6 (18 sequences), IGHV10 (6 sequences), IGHV17(ORF) (35 sequences), and IGHV36 (9 sequences) were identified within group 2. These IGHV segments were distributed between the isotypes in similar proportions. Only the segment IGHV10 was identified in all group 3 sequences. The phylogenetic alignment of IGHD segments revealed dissimilarities between recombined and genomic sequences, which resulted in low sequence identities. In addition, there were homologies between the germline IGHD segments ranging from 81.4% (IGHD2 and IGHD8) up to 97.6% (IGHD1 and IGHD6). Therefore, it was not possible to unambiguously annotate all CDR3H regions of the sequences analyzed. Nevertheless, IGHD2 was preferentially recombined in sequences possessing exceptionally long CDR3H. At the IGHV-IGHD junctions, conserved short nucleotide sequences (CSNS) were identified, which are rich in A nucleotides. The inclusion of N and P nucleotides was noted within the IGHD-IGHJ junction. In addition to the presence of CSNS in sequences possessing very long CDR3H, these CSNS were also discovered in sequences with CDR3H of mid-length. IGHD4 was used most often in these group 2 sequences. All other known IGHD segments were recombined less frequently in this group. The short IGHD segments IGHDQ52 and IGHD4(1 and 2) – possessing 15 and 36 nucleotides – were identified in very short CDR3H regions.

## Discussion

The *in silico* analyses of the genomic organization of the heavy-chain locus revealed differences from previous mapping and annotation results. The functional locus was mapped to BTA21q23-q24 [21], [22], [23], where we also detected ten variable and two diversity segments together with the exons coding for IgM(F), IgE, and IgA(F). The order of the respective segments highlighted deviations from other fully described mammalian loci [16], [52]. Zhao et al. [21] have already described the constant heavy-chain locus by means of BAC clone analysis, as well as the joining segments organized upstream of IgM. These BAC clones were not introduced into the genomic assembly. Since the UMD2 assembly used mapping data, synteny with the human genome, and paired-end sequence information [53], we did not expect heavy-chain loci on BTA7, BTA8, and BTA20. Upstream of IGHV11Ψ, the bovine contig-NW_003064289 on BTA7-harbors the genes for FBXL12 (F-box and leucine-rich repeat protein 12), UBL5 (ubiquitin-like 5), PIN1 (peptidylprolyl cis/trans isomerase, NIMA-interacting 1), and OLFM2 (olfactomedin 2) which share homology with HSA19. The human gene following downstream is COL5A3 (collagen, type 5, alpha 3). No variable segment was found between OLFM2 and COL5A3 on HSA19p13.2. The bovine pseudo δ-chain locus on BTA7 revealed no human equivalent. The human 5pter part is inversely syntenic to BTA20qter [54]. On contig NW_003104522.1, we found the gene ADCY2 (adenylat cyclase 2) as an anchor. The bovine IgG2(ORF) (IGCGAMMA) gene, annotated between AHRR (aryl-hydrocarbon receptor repressor) and PDCD6 (programmed cell death 6), has no homologue on the HSA5pter, although it has been noted that AHRR and PDCD6 overlap on HSA5pter. For IGHD4 to 7, and pseudo δ-chain on BTA8, no specific genes were identified. Human syntenic groups on BTA8 were described for HSA8p and HSA9 [54]. No additional genes were determined on contig NW_003066919, with a complete IgDΨ, IgM(ORF) gene and IGHJ1(ORF) to 6(ORF), as well as IGHDQ52(ORF). Hybridization investigations assigned an IgM-like chain (probe IGHML1) to the syntenic group U16; corresponding to HSA9q [24], [55]. Later, IGHML1 was assigned to BTA11q23 by hybridization [24], [25], which was supported by the detection of six IGHJ segments on the same chromosome using BAC clone and locus-specific PCR analysis [26]. We were not able to identify an IgM-like locus on BTA11. Based on these results and the fact that we observed a transcribed IGHV from a putative orphon, we concluded an incorrect and incomplete annotation of the bovine immunoglobulin heavy-chain locus, which may be solved by the re-sequencing of the described localizations and underpinned by different authors and methods.

Previous studies have already classified bovine IGHV segments into clan II, with the closest homology to the human VH2 family [6], [12], [13], [44]. Quite in contrast to the statement made by Tutter and Riblet [28] and Berens et al. [6], none of the genomic sequences clustered with the human clan III family VH3. All of the functional bovine IGHV segments are most closely related to the human VH2 family represented by IGHV2-05, which explains the exclusive transcription of only one bovine VH family [6], [13], [56]. The comparison with human sequences was performed by Saini et al. [12] using one VH4 family sequence only. The second bovine VH family described here consisted only of pseudogenes and clustered with the human VH4 and VH6 family. We were now able to describe the second bovine VH family (boVH2) previously proposed [6], but we had no indications of the possible gene conversions using boVH2 segments in the sequences investigated, as shown for the bovine λ-light chains [15]. We would propose that IGHV5Ψ and IGHV8Ψ should be assigned to the boVH2 family as they are fragmented loci and share 70.1% sequence identity with IGHV1Ψ, IGHV18Ψ, IGHV27Ψ, and IGHV30Ψ.

With regard to the description of unusually long CDR3H in bovine IgM, long and short IGHD segments have already been described. Independent of nucleotide addition during rearrangement they contribute directly to CDR3H length heterogeneity. Nevertheless, the genomic IGHD segments showed high homologies among themselves, which resulted in the complicated annotation of the transcribed IGHD. In particular, many hypermutations within the recombined IGHD segments led to low sequence identities. Intrinsic hot spots as targets for somatic hypermutations within CDR1H, CDR2H, and CDR3H were already found in a bovine fetus. Furthermore, CDR3H length heterogeneity, junctional flexibility, and somatic hypermutation are thought to contribute solely to IgM antibody diversification in both bovine fetus and adult cattle [32]. As we found CDR3H length heterogeneity in all isotypes, exceptionally long CDR3H are apparently not primarily generated to compensate the restricted flexibility of IgM caused by reduced Pro numbers within Cµ2. The most recent study on the IgG repertoire in calves also showed very long CDR3H [35]. Therefore, antigen selection of variable domains and class switch recombination seem to be of higher impact. We did not observe any evidence to suggest that the combination of two different IGHD segments enhances diversity, which was the case for horses [16]. The exceptionally long CDR3H were generated by the direct fusion of a single IGHV segment (IGHV10), the longest IGHD segment (IGHD2), and one functional IGHJ segment (IGHJ1), as described previously [30]. In very short CDR3H, we noted a preferred use of the short IGHD segments, IGHDQ52 and IGHD4. In sequences of CDR3H group 1 and 2, no predominant use of one special IGHV segment was determined. According to structural analyses, spatial distances are not thought to contribute to preferred IGHV-IGHD-IGHJ rearrangements, as there are conformational changes of chromatin resulting in the repositioning of the IGHD cluster and the merging of proximal and distal IGHV regions during early B cell development [57]. All IGHV segments identified were found to be functional. Thus, there is no evidence for gene conversion in bovine immunoglobulin heavy chains, which is already known to contribute to the diversity of chicken immunoglobulin heavy chains and bovine λ-light chains [15], [58]. Conserved short nucleotide sequences (CSNS), which were inserted into the IGHV-IGHD junction, were found both in intermediate length and very long CDR3H. This novel mechanism, which contributes to antibody diversification, is neither restricted to immunoglobulin heavy chains with exceptionally long CDR3H, nor is it isotype restricted. As the insertion of CSNS is supposed to directly follow antigen exposure during the development of an immune response [30], we conclude class switch recombination to be responsible for isotype-independent, long CDR3H in cattle. In addition, exceptionally long CDR3H protrude from the variable domain with support from the λ-light chains. Thus, there is no conventional combining site and the other two CDRH do not contribute to antigen binding. Instead, this function is undertaken by side chains that are exclusively contained within long CDR3H regions, as investigated by structural comparisons with protein toxins [37].

Transcribed CDR1H, CDR2H, and short CDR3H sequences showed both hydrophilic and hydrophobic amino acid residues. In long CDR3H, hydrophilic amino acid residues were represented mainly by repetitive Gly, Tyr, and Ser, whereas Gly dominated in most of the sequences analyzed – which is consistent with previous findings [27]. The major usage of the hydrophilic reading frame was already described in humans [59], mice [60], chicken [58], and rabbits [61]. Their occurrence in antigen-binding loops is thought to enhance flexibility and recruit somatic hypermutations for advantageous antigen binding [59].

Moreover, in accordance with descriptions provided previously, we identified multiple and mainly even numbered Cys within exceptionally long CDR3H, which were accumulated in the middle of the CDR3H [31], [33]. These Cys are predicted to form intra and inter CDRH disulfide bonds, rigidifying the combining site or helping to stabilize long CDR3H, as demonstrated in the crystallized human Fab Kol [62] and the camel cAb-Lys3 single domain antibody [63]. In this context, additional Cys in CDR2H were noticed before, when there were only one or three Cys in CDR3H [31]. Concerning the IMGT numbering system, we solely identified one additional Cys in CDR2H, when one Cys in CDR3H was found in two sequences of IgE with middle-length CDR3H. When numbering the amino acids according to Kabat et al. [8], ten sequences classified in group 2 possessed one Cys in CDR3H and showed an additional Cys in CDR2H. We did not note analogues for sequences from group 1 or group 3. In contrast to the findings that there is at least one Cys in CDR3H regions containing more than 12 amino acid residues, and that there is no Cys if the CDR3H possessed less than 10 amino acid residues [27], we also observed CDR3H sequences of intermediate length without Cys residue in CDR3H.

We annotated the bovine immunoglobulin heavy-chain locus, demonstrated the expression of unusually long CDR3H in the five bovine immunoglobulin isotypes, and specified their genomic origin. Thus, this study reviewed the opinion that exceptionally long CDR3H are a unique feature of bovine IgG1-3 and IgM.

### Note

Sequences can be found under GenBank Acc.No. KC471523 to KC471622.

## Supporting Information

Table S1 **Genomic annotation of the bovine immunoglobulin heavy-chain locus.** For a functional gene (F), the complete coding sequence, octamer motif, TATA box, splicing signals or recombination signal sequences (RSS), and poly A motif were identified. Putative functional genes ((F)) lacked some of the described parts due to end of the contig or N’s introduced into gaps for example. ORFs are classified by alterations in the splicing signals, RSS or regulatory elements. The extension (ORF) describes fully functional genes in putative orphons. Pseudogenes (Ψ) revealed stop codons, frameshifts or mutations within the RSS, which lead to abolition of recombination. Fragmented loci were also defined as Ψ [38], [39], [40], [41].
(XLS)
